# Pathologic complete response after Sintilimab combined with FOLFOX therapy in MSI-H type patients with locally advanced GC: a case report

**DOI:** 10.3389/fonc.2025.1560450

**Published:** 2025-05-08

**Authors:** Xiaoke Wang, Yuanhui Gu, Lin Yi, Tao Wu, Ling Wang, Jiao Tian, Yuyuan Lu, Penghui Jin, Xin Yang, Yan Yang

**Affiliations:** ^1^ Department of General Surgery, Gansu Provincial Hospital, Lanzhou, Gansu, China; ^2^ First School of Clinical Medical, Gansu University of Chinese Medicine, Lanzhou, Gansu, China; ^3^ School of Traditional Chinese and Western Medicine, Gansu University of Chinese Medicine, Lanzhou, Gansu, China; ^4^ Pathology Department, Gansu Provincial Hospital, Lanzhou, Gansu, China; ^5^ Pharmacy Department, Gansu Provincial Hospital, Lanzhou, Gansu, China

**Keywords:** advanced gastric cancer, MSI-H, PCR, immunotherapy, case report

## Abstract

As one of the most common gastrointestinal tumors, Gastric Cancer (GC) poses a serious threat to human health due to its high morbidity and mortality. The current treatment strategy is a comprehensive treatment program mainly based on surgery, especially for advanced GC patients. The emergence of immune checkpoint inhibitors has completely changed this status quo, and the synergistic effect of neoadjuvant immunotherapy combined with chemotherapy has significantly improved the resection and radical rate and overall survival of patients with advanced local GC. We present a case of locally advanced GC (cT4N0Mx) with microsatellite instability high (MSI-H) and PD-L1 Combined Positive Score (CPS)=2. The patient received neoadjuvant therapy with Sintilimab combined with FOLFOX (folinic acid (leucovorin), 5-fluorouracil (5-FU), and oxaliplatin), and significantly reduced tumor volume after 3 cycles of treatment. Then she underwent subtotal gastrectomy with gastrojejunostomy and D2 lymph node dissection. The postoperative pathological results showed that no cancerous tissue remained in the tumor tissue, and pathologic complete response (pCR) was achieved. The first cycle of adjuvant therapy with the same protocol was received after surgery. During adjuvant therapy, patients mainly experienced side effects such as dyspepsia, nausea and mild myelosuppression. Therefore, immunotherapy with Sintilimab combined with FOLFOX chemotherapy has the potential to be an effective treatment option for patients with resectable locally advanced MSI-H GC.

## Introduction

Gastric cancer (GC) is defined as a primary epithelial malignancy originating in the stomach with multiple risk factors. GC is the fifth most common cancer in the world and the fifth leading cause of cancer-related death, with more than 650,000 people losing their lives every year due to GC, a serious threat to the health of all mankind (1). For early GC, endoscopic or surgical resection is the main treatment means. However, due to the lack of typical clinical Pointers for early GC, many GC patients have already developed to the advanced stage when they are found. Coupled with the lack of effective treatment, the prognosis is often poor, and the life expectancy of most patients is only 1 year (2, 3). Therefore, advanced GC patients need chemotherapy, radiotherapy and molecular targeted combination therapy. Due to the continuous exploration of tumor immune microenvironment (TIME) in recent years, immunotherapy for GC has been greatly promoted. Immunotherapy is superior to conventional therapies in terms of efficacy and tolerability (4) and has therefore been extensively studied in GC, especially since different subpopulations have been identified based on microsatellite status.

Locally advanced GC (LAGC) is clinically defined as disease with a tumor stage of T3-T4 and/or regional lymph node positivity. Among them, microsatellite instation-high (MSI-H)/deficient mismatch repair (dMMR) GC patients are relatively rare and a unique subtype, accounting for only 6% of advanced GC cases (5). Microsatellite instability (MSI) refers to the high mutability of repeated sequences (called microsatellites) scattered throughout the genome (6). MSI-H is derived from dMMR, and since dMMR can cause high mutation of tumor cells and produce multiple neoantigens, the number of infiltrating lymphocytes and the expression of intratumoral immune checkpoints in MSI-H cancer are much higher, which is highly immunogenic. The use of anti-programmed cell death 1 (PD-1) or programmed cell death ligand 1 (PD-L1) antibodies is an effective treatment regimen (7, 8).

Neoadjuvant therapy is an effective treatment for GC, and its purpose is to reduce the volume of the primary tumor to facilitate resection, thereby reducing the local recurrence rate, reducing the spread of tumor cells during resection, and improving the overall survival rate (OS) of GC patients (9, 10). Studies have found that T cells proliferated more strongly before surgery, resulting in significantly higher levels of CD8+T cells in both peripheral blood and organs. Therefore, immunotherapy as a neoadjuvant therapy is more biologically effective than adjuvant therapy (7). Especially for patients with GC such as MSI-H, there is currently no Grade I recommendation for first-line therapy. In 2023, the Chinese Society of Clinical Oncology (CSCO) only includes pembrolizumab monotherapy as a secondary recommendation for first-line therapy (11). The potential benefits of perioperative addition of molecularly targeted drugs or immune checkpoint inhibitors are still being investigated. In this case, we report on perioperative Sintilimab combined with FOLFOX in a patient with locally advanced MSI-H GC with a CPS of 2.

## Clinical information

**Figure 1:**
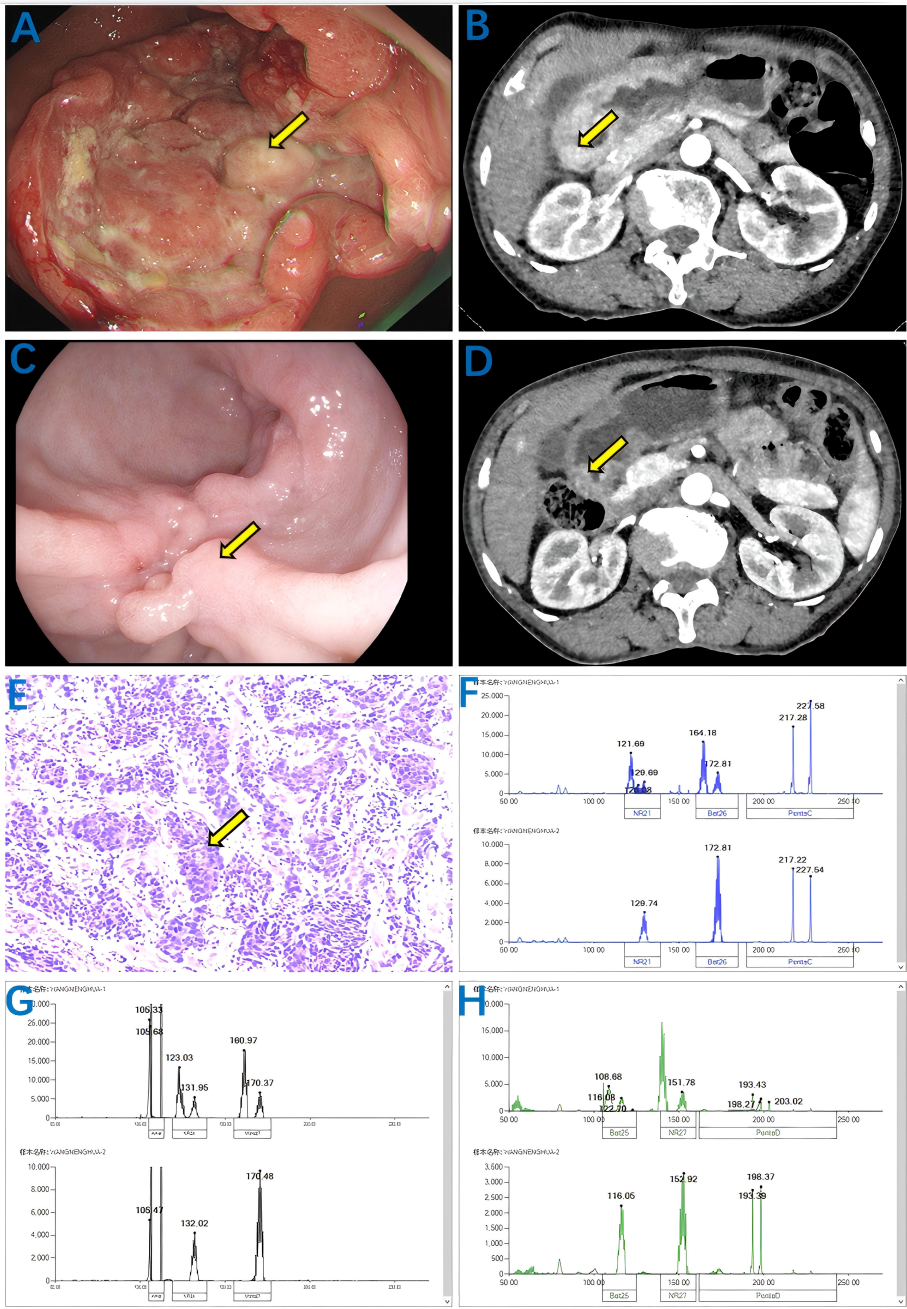
Results of abdominal CT, gastroscopy, biopsy and MSI status were enhanced before and after treatment with Sintilimab combined with FOLFOX. Before treatment: **(A)** Gastroscopy showing antrum mass (yellow arrow); **(B)** Enhanced abdominal CT showing antral carcinoma (cT4N0Mx) (yellow arrow); **(E)** Biopsy pathology showing moderately to poorly differentiated adenocarcinoma (yellow arrow); **(F–H)** MSI status indicates that microsatellite loci BAT25, BAT26, NR-21, NR-24, NR-27, MONO-27 are unstable, namely MSI-H. After treatment, the volume of the mass decreased significantly: **(C)** the gastroscope showed a mass of the gastric antrum (yellow arrow); **(D)** Enhanced abdominal CT showing antral carcinoma (cT4N0Mx) (yellow arrow).

On August 29, 2024, a 67-year-old female patient presented with upper abdominal distension and dyspepsia for half a month. Gastroscopy revealed a huge ulcer-like mass visible on the stomach curvature and posterior wall at the gastric antrum. The ulcer base was uneven, the surface was covered with dirty moss, and the edge of the ulcer was raised like a levee. The final diagnosis was antral carcinoma, gastric retention, and chronic atrophic gastritis (**Figure 1A**). For further evaluation and treatment, the patient was admitted to the Department of General Surgery of Gansu Provincial People’s Hospital. Abdominal physical examination showed no abnormality, height 150 cm, weight 45 kg, BMI 20 kg/m2, ECOG-PS 1; In our hospital, whole-abdominal enhanced CT (**Figure 1B**) showed antrum carcinoma (cT4N0Mx), Borrmann type III. Gastroscopic pathological biopsy results showed medium-poorly differentiated antrum adenocarcinoma (**Figure 1E**). Immunohistochemistry showed Her-2 (-), PD-1 (-), HER-2 (-) and PD-1 (-). PD-L1 (SP263) (CPS:2%),P40 (-), CgA (-), Syn (-), Ki67 (hot spot 80%). In order to develop a more accurate neoadjuvant treatment strategy, we further evaluated the MSI/MMR status. The results showed that the six microsatellite loci BAT25, BAT26, NR-21, NR-24, NR-27 and MONO-27 were unstable, namely MSI-H (**Figures 1F–H**). The results of related tumor markers showed that alpha-fetoprotein was 35.90 ng/ml (normal <8.78ng/ml) and carcinoembryonic antigen was 17.51 ng/ml (normal <5 ng/ml). Due to the large tumor size and high surgical risk, the patient was diagnosed as medium-low differentiated antral adenocarcinoma with cT4N0Mx after discussion by our multidisciplinary team (MDT). Therefore, we developed a treatment regimen of sindilizumab 135 mg, once every 3 weeks on day 1; Oxaliplatin 100 mg, every 3 weeks on day 1, calcium folinate 500 mg, every 3 weeks on day 1, fluorouracil 3200mg continuous infusion for 46 hours, every 3 weeks. During this period, patients experienced nausea (CTCAE grade 1), vomiting (CTCAE grade 1), fatigue (CTCAE grade 1), and myelosuppressive reactions, including leukopenia (CTCAE grade 1) and thrombocytopenia (CTCAE grade 1). After three cycles of this regimen, gastroscopy and whole-abdominal enhanced CT showed a significant decrease in the tumor lesion area compared with the previous one (**Figures 1C, D**).

**Figure 2:**
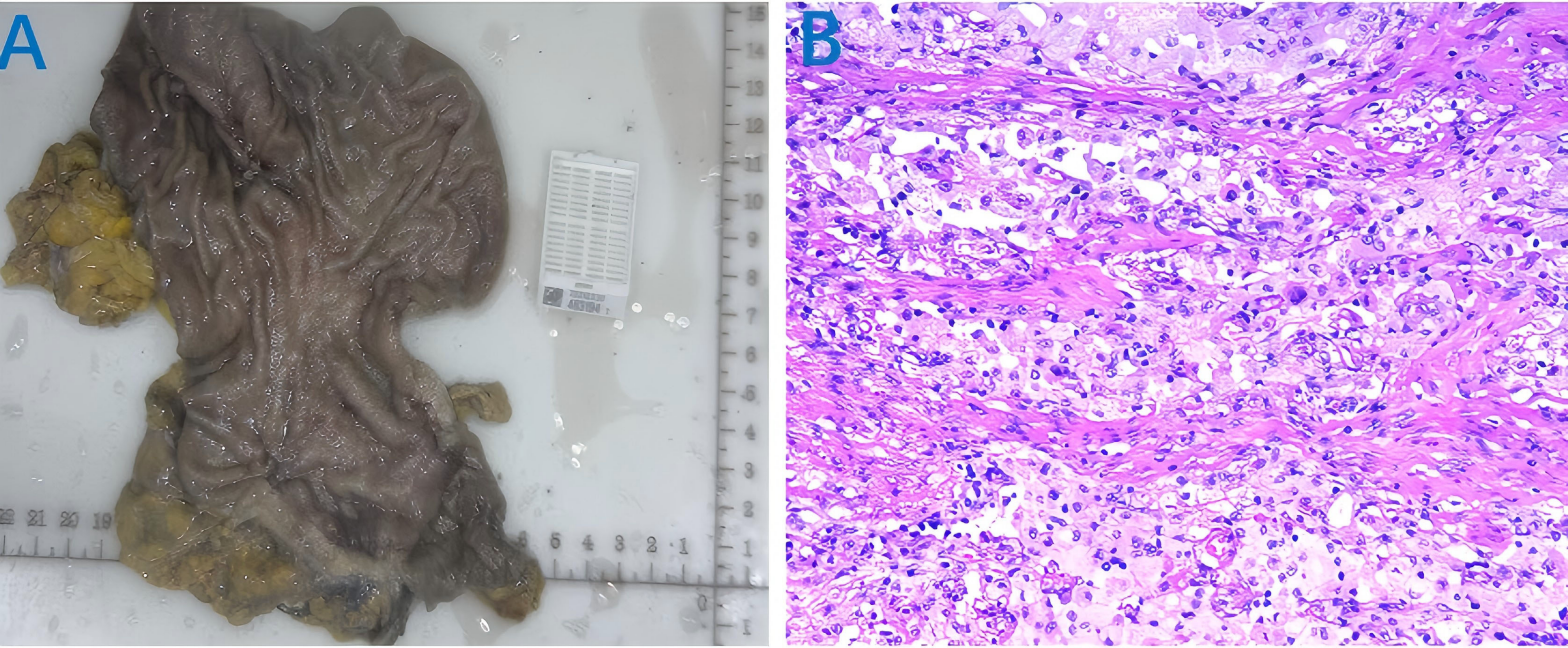
Postoperative tumor tissue specimen and pathological examination results. **(A)** tumor tissue specimens; **(B)** Postoperative pathological examination showed TRG: grade 0.

**Figure 3:**
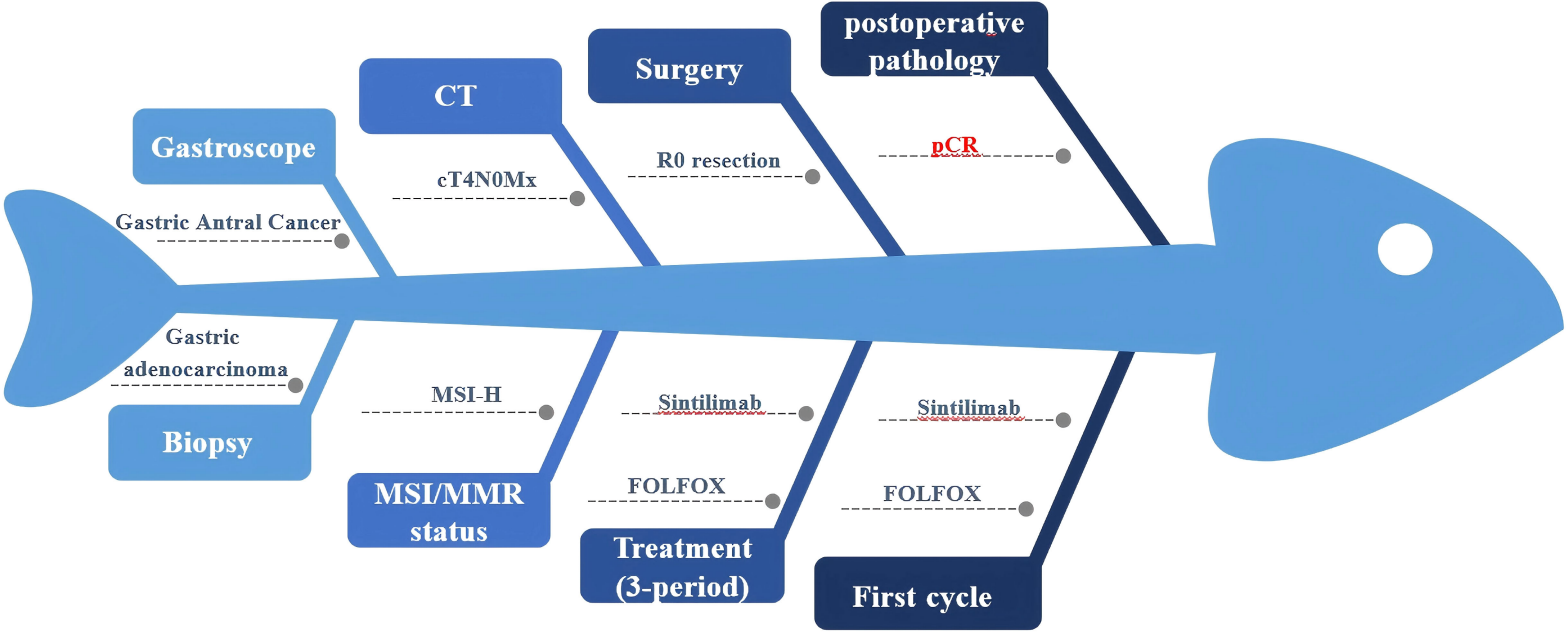
Fishbone diagram of the patient during the whole course of perioperative treatment. MSI/MMR, Microsatellite instability/Mismatch repair. FOLFOX, Folinic acid (leucovorin); 5-fluorouracil (5-FU), and oxaliplatin. pCR, Pathologic complete response.

On October 29, 2024, after discussion by our multidisciplinary team (MDT), the patient underwent laparoscopic subtotal gastrectomy with gastrojejunostomy and D2 lymph node dissection (**Figure 2A**). The postoperative pathological report showed that there was no residual cancer tissue in the tumor tissue, low grade intraepithelial neoplasia in the focal mucosal glands, and no cancer tissue involvement in the upper and lower incisal margins and the greater omentum. No metastatic cancer 0/28 was found in perigastric lymph nodes (0/11 in the lesser curvature of the stomach and 0/17 in the greater curvature of the stomach). The pathological evaluation criteria for AJCC/CAP after neoadjuvant chemotherapy were as follows: TRG: grade 0, that is, no cancer cells were found (complete regression) and pathological complete response (pCR) was achieved (**Figure 2B**). On November 19, 2024, the patient underwent the first cycle of postoperative adjuvant therapy with the same regimen, during which only mild myelosuppression occurred as a side effect. Up to now, the patient has undergone perioperative comprehensive treatment for more than 2 months, and the tumor tissue has successfully achieved pCR, with significant curative effect (**Figure 3**).

## Discussion

Cytotoxic drugs, including fluoropyrimidine, platinum, taxoids and irinotecan, are the main adjuvant therapy for advanced GC. Perioperative treatment for GC (neoadjuvant chemoradiotherapy + surgery + adjuvant chemotherapy/chemoradiotherapy) in Western countries has been proven to be superior to surgical treatment alone in terms of improving the radical cure rate (12). Neoadjuvant therapy before radical GC resection has significant advantages in improving tumor remission rate and R0 resection rate (13, 14). However, perioperative treatment has not been shown to be effective in MSI‐HGC patients (15, 16), and the results of two clinical trials, MAGIC and CLASSIC, have shown that MSI‐HGC patients do not benefit from perioperative or postoperative chemotherapy (15, 17). Similarly, a meta-analysis of four large randomized clinical trials (MAGIC, CLASSIC, ARTIST, and ITACA-S) showed no significant benefit in OS at 5 years in the postoperative adjuvant chemotherapy group compared with the surgery group (18). This is because MSI-H and dMMR increase the production of somatic mutations and neoantigens, often leading to extensive lymphocyte infiltration and elevated expression of immune checkpoints in the tumor microenvironment (TME), and therefore, MSI-H/dMMR patients are less responsive to cytotoxic drugs and highly sensitive to immunotherapy (19). As shown in the case - series study by Liu et al. (20), pembrolizumab combined with neoadjuvant chemotherapy may benefit locally - advanced MSI - H gastric cancer patients. Of the 6 such patients,3 achieved a pathological complete response (pCR).

Immunotherapy has been extensively studied in gastrointestinal cancers in recent years, and immune checkpoint inhibitors (ICIs) (either monotherapies or in combination with other therapies) have shown anti-tumor effects in a variety of solid tumors, including gastrointestinal tumors (21). Currently, anti-programmed cell death protein-1/programmed death ligand 1 (PD-1/PD-L1) antibodies are mainly approved for the treatment of unresectable or metastatic solid tumors (9). The success of Chekmate-649, ORIENT-16, Keynote-177, Keynote-590 clinical trials has made anti-PD-1/PD-L1 antibody combined with chemotherapy has become the primary first-line treatment for GC (GC), mismatch repair defect (dMMR)/high microsatellite instability (MSI-H) colorectal cancer (CRC) (19). In addition, the INFINITY study showed that Tremelimumab combined with Durvalumab, as a new adjuvant therapy without chemotherapy, showed good activity and high pCR rate in mismatch repair defect/MSI-H gastric cancer or gastroesophageal conjunctive adenocarcinoma (22). In addition, The GERCOR NEONIPIGA Phase II Study showed that neoadjuvant opdivo combined with low-dose ipilimumab was feasible in dMMR/MSI-H resectable gastric cancer or GEJ adenocarcinoma patients, without unexpected toxicity, and achieved a high pCR rate (23).

Results of two trials, CheckMate 64954 and ORIENT-1655, proved that nivolumab or Sintilimab combined with chemotherapy is safer and more effective than chemotherapy alone as first-line treatment (24, 25). Sintilimab is an intravenously available anti-PD-1 monoclonal antibody with a stronger PD-1 affinity compared to the immune checkpoint inhibitors nivolumab and pembrolizumab. In a single-arm, phase II HER2-negative locally advanced GC study, the R0 removal rate of Sintilimab combined with fluorouracil, leucovorin, oxaliplatin, and docetaxel (FLOT) was 93.1%. objective response rate (ORR) was 55.2%, pathological complete response (pCR) was 17.2% (26). Another ORIENT-16 randomized clinical trial in China showed that for GC patients with CPS ≥5, the median overall survival in the Sintilimab combined chemotherapy group was 18.4 months, which was significantly better than 12.9 months in the placebo group (27). In the phase 2 study in which the treatment of Sintilimab combined with oxaliplatin and capecitabine (CapeOx) can remove advanced GC, the R0 removal rate was 97.2%, and the objective response rate (ORR) was 47.2%. pathological complete response (pCR) was 19.4% (9).

As the relationship between PD-L1 expression profile and patient prognosis, MSI status and the efficacy of neoadjuvant immunotherapy has been controversial (28), the optimal treatment plan for MSI-H/dMMR tumors, whether it is ICI monotherapy or ICI combined with chemotherapy, is still uncertain (29). MSI-H/dMMR type tumors can stimulate the expression of PD-1/PD-L1 (30), and in GC, PD-L1 is frequently overexpressed on tumor cells and immune cells in MSI-H/dMMR tumor tissues (31–33). For MSI-H patients with PD-L1 CPS ≥5, the 3-year OS benefit is greater after immunotherapy combined with chemotherapy (34). In a single-arm Phase II exploratory trial (NCT03878472), ICI based neoadjuvant/conversion therapy has good efficacy and feasibility in cT4a/bN+ GC. Especially for MSI-H and PD-L1 positive patients (35). An evaluative study of KEYNOTE-059, KEYNOTE-061, and KEYNOTE-062 found that patients with advanced GC with MSI-H benefited significantly from first - to third-line pembrolizumab monotherapy (36). However, for GC patients with PD-L1 CPS <5, immunotherapy combined with chemotherapy did not show the desired therapeutic effect, and the outcome was consistent with chemotherapy alone (27, 37). In patients with MSI-H tumors and PD-L1 ≥1 in the Phase III KEYNOTE-062 trial, pembrolizumab monotherapy showed a more favorable OS trend compared to chemotherapy alone (HR, 0.29; 95% CI 0.11-0.81), while pembrolizumab combined with chemotherapy was slightly less effective (HR, 0.37; 95% CI 0.14-0.97), in contrast, pembrolizumab combined with chemotherapy had better ORR and PFS than pembrolizumab monotherapy (36, 38).

As mentioned above, it has been found in previous studies that immunotherapy combined with chemotherapy, including Sintilimab, can achieve a good effect in GC patients with MSI-H and PD-L1 ≥5. However, for patients with MSI-H and PD-L1 CPS <5, there is still debate on this diagnosis and treatment strategy (39). Msi-h has a promising application prospect as a biomarker of GC perioperative immunotherapy efficacy (11, 40). The National Comprehensive Cancer Network (NCCN) clinical practice guidelines recommend universal detection of MSI or MMR for all newly diagnosed GC patients (41). To identify patients who need immunotherapy. In this case, we successfully reported a patient with MSI-H and PD-L1 CPS=2. Due to the patient’s good clinical response before surgery, the patient’s abdominal pain was eased, body weight was increased, and tumor markers were significantly decreased. The treatment strategy of Sintilimab combined with FOLFOX every three weeks was adopted to make the tumor tissue reach pCR successfully. Due to the short follow-up time, it is not possible to determine whether the results are attributable to immunotherapy or chemotherapy, so it is unclear how this response translates into long-term outcomes, so the strength of conclusions about its efficacy is limited and will await our further follow-up.

In summary, perioperative treatment focusing on surgery is particularly important for patients with locally advanced resectable GC. PD-L1 CPS and MSI/MMR status should be evaluated for all patients with advanced GC, especially before neoadjuvant therapy. For patients with MSI-H and PD-L1 CPS<5, immunotherapy combined with chemotherapy not only improves the R0 resection and pathological response rate of GC patients, but also improves the survival time of patients. Our study provides a good basis for the feasibility and safety of perioperative immunotherapy combined with chemotherapy and immunotherapy in this type of GC patients, and also shows that immunotherapy can provide a new prospective treatment option.

## Data Availability

The original contributions presented in the study are included in the article/supplementary material. Further inquiries can be directed to the corresponding author.
